# Effects of Natural Products on Bacterial Communication and Network-Quorum Sensing

**DOI:** 10.1155/2020/8638103

**Published:** 2020-05-24

**Authors:** Min Yang, Fanying Meng, Wen Gu, Fengjiao Li, Yating Tao, Zhengyang Zhang, Fan Zhang, Xingxin Yang, Jingping Li, Jie Yu

**Affiliations:** College of Pharmaceutical Science, Yunnan University of Chinese Medicine, 1076 Yuhua Road, Chenggong District, Kunming, Yunnan Province, China

## Abstract

Quorum sensing (QS) has emerged as a research hotspot in microbiology and medicine. QS is a regulatory cell communication system used by bacterial flora to signal to the external environment. QS influences bacterial growth, proliferation, biofilm formation, virulence factor production, antibiotic synthesis, and environmental adaptation. Through the QS system, natural products can regulate the growth of harmful bacteria and enhance the growth of beneficial bacteria, thereby improving human health. Herein, we review advances in the discovery of natural products that regulate bacterial QS systems.

## 1. Background

Until the 1980s, it was believed that bacteria individually grow and multiply with no communication between cells [1]. It is now accepted that a common exchange of information exists between bacteria and that individuals of the same or different species compete or cooperate through quorum sensing (QS). In this review, we will discuss the underlying molecular mechanisms of QS and highlight its role in human health. We then summarize the regulatory role of natural products in QS systems and probe their potential value in disease prevention, diagnosis, and disease treatment.

## 2. Regulatory Mechanisms of Quorum Sensing

In 1970, the marine bacteria *Photobacterium fischeri* was found to secrete a substance that controlled the luminescence of cells [2]. Subsequent studies revealed that this substance regulated bacterial density. The bacterium releases signaling molecules (AI, autoinducers) that stimulate the bioluminescence system at a high population density [3]. This environmental sensing mechanism is also common to other marine organisms [4]. In the 1980s, scientists identified the bioluminescence producing gene-Luminescence (*lux*) in *Vibrio fischeri*, and subsequently, the AI in *Photobacterium fischeri* as an *N*-(3-oxohexanoyl)-DL-homoserine [5–7]. This laid the material foundation for QS. In 1994, Fuqua first proposed the concept of QS, in which bacterial phenotypes are regulated according to the concentration of chemical signals produced by individuals or colonies of bacteria [8].

Bacterial QS from a medical perspective had received increasing research focus. QS regulates the microbe balance, which is closely linked to the occurrence and development of diseases such as intestinal inflammation [9], pneumonia [10], immune system [11], and even metabolic syndrome [12, 13].

### 2.1. Quorum-Sensing System

Bacteria release chemical AIs through the action of QS that accumulate within the bacterial environment. Through detecting changes in AI concentrations, bacteria exchange information. A range of signaling molecules related to bacterial QS has been discovered. Dependent on the type of signaling and sensor systems, the QS of bacteria can be classified. In Table 1, we summarize the pathways associated with each QS system and its distribution in bacteria [14–33].

(1) Acyl-homoserine lactone (AHL) was the earliest discovered signaling molecule produced by Gram-negative bacteria. A typical feature of AHL molecules is the inclusion of homoserine lactone rings and *N*-acyl chains. The AHL produced by bacteria is of varying lengths and side-chain modifications [14].

(2) Gram-positive bacteria produce autoinducing peptides (AIP) as signaling molecules. Precursor peptides are synthesized in the ribosome, and the precursor peptides undergo a series of modifications during outward transport leading to the formation of mature AIP. Due to the complexity and specificity of the synthetic process, the structural information on AIP is sparse [19, 23].

(3) LuxS/autoinducer-2 (LuxS/AI-2) was first discovered in the marine bioluminescent bacterium *Vibrio harveyi*, with its active structure being furanosyl borate diester [34]. Studies show that LuxS/AI-2 is widely distributed in Gram-negative and Gram-positive bacteria, forming specific signaling molecules for intra- and interspecies exchange. This is generally considered a universal signaling molecule for bacterial communication.

(4) In addition to the described signaling pathways, other signaling molecules have been discovered, including AI-3 [29], diffusible signal factor (DSF) [30], and Pseudomonas quinolone signal (PQS) [30].

QS promotes the exchange of information between individuals through signaling molecules, thereby triggering the expression of a series of genes to complete QS mechanisms. In Gram-negative bacteria, one species bacterial QS system often contains multiple structures AHL molecules. Moreover, there are differences in the structure of AHL molecules in different species of bacteria, and the quorum-sensing mechanism is also different. Therefore, AHL molecules are often used for intraspecific communication of Gram-negative bacteria. AIP exists in the quorum-sensing system of Gram-positive bacteria, and each kind of bacteria secretes unique AIP that different from other bacteria. Therefore, the QS system mediated by AIP is a way of intrabacterial communication. AHL molecules have not been found in Gram-positive bacteria. The AI-2 signal molecule can be synthesized by a variety of bacteria, including Gram-positive and Gram-negative bacteria, so AI-2 can be used for interspecific communication.

In addition, multiple QS systems are present in the same bacteria. For example, two interrelated QS systems (Las and Rhl) and PQS systems in *Pseudomonas aeruginosa*, SdiA, Lsr, and AI-3 QS are present in *E. coli*, whilst LuxS/AI-2 systems have been identified in *Staphylococcus aureus*. Whether these regulatory systems in bacteria interfere with other processes requires further investigation. In addition, other microbial QS systems are still to be identified, and their identification forms a key aspect of QS systems.

### 2.2. Quorum-Quenching Systems

There is evidence that QS allows bacteria to harmonize their behavior. Accordingly, organisms have developed strategies to counteract QS. Such mechanisms are termed quorum quenching (QQ) [35].

QQ disrupts the communication between bacteria, thereby inhibiting group behaviors, including the production of virulence factors. QQ is now known to play a role in the competitive inhibition and degradation of signaling molecules through (1) inhibition of signaling molecule generation: The QS process is inseparable from the generation and participation of signaling molecules. By inhibiting related enzymes in the signaling molecule synthesis pathway, the generation of signal molecules can be blocked and QS can be inhibited. For example, Triclosan can inhibit enoyl-ACP reductase (an important protein in the AHL generation process) [36]. (2) Competitive inhibition: By synthesizing some structural analogs of signaling molecules, they can competitively bind with corresponding receptor proteins, and block the binding of signal molecules to receptors, thereby affecting the transmission of signal molecules. For example, halogenated furanones (AHL structural analogs) can inhibit quorum sensing [37]. (3) Degradation signal molecules: By using degradation enzymes to degrade signal molecules, the concentration of signal molecules is lower than the threshold, thereby destroying the QS system. For example, MacQ is an AHL acylase which can mediate QQ [38].

In summary, bacteria prevent the accumulation of signaling molecules through QQ, thereby blocking signal exchange and the expression of QS-related genes. Bacteria adjust QS and QQ to maintain the microecological balance of organisms that regulate group behavior. On the one hand, exploring the relationship between bacterial QQ and QS provides new avenues of prevent and treat bacterial disease.

## 3. Quorum Sensing and Quorum Quenching in Human Health

Bacteria are essential components of the human ecosystem. Bacteria are abundant on the surface of the human body, mouth, respiratory tract, intestine, and vagina. The total numbers of bacteria in healthy individuals exceed 100 trillion and achieve a dynamic balance through a network of interactions between microbes. When this balance is broken, pathogenic infections, loss of immunity, and inflammation result. Herein, we summarize the relationship between bacterial QS and human health, providing new directions for the treatment of disease.

### 3.1. Quorum Sensing Mediates the Probiotic Properties of Bacteria

Probiotics are taken to improve immune health, but their effects on QS are poorly defined. *Bifidobacterium* that inhabits the mammalian intestinal tract is known to improve human health. LuxS/AI-2 QS systems have been identified in *Bifidobacterium*, and QS-signaling molecules including AI-2 promote biofilm formation in *Bifidobacterium* [39]. Carbohydrates (mannose, fructose, sucrose, and lactose) significantly improve the secretion of AI-2 from the *Bifidobacterium*. The concentration of AI-2 has increased by 4.49~89.45% following the addition of carbohydrates to the *Bifidobacterium* [40]. Mice infected with Shiga toxin-producing *Escherichia coli* (STEC) O157:H7 show strong anti-infective activity following the intake of the *Bifidobacterium breve* strain in Yakult. Further studies have shown that *B. breve* produced high concentrations of acetic acid (56 mM) and lower intestinal pH (to pH 6.75), inhibiting the production of STEC O157:H7 toxin Stx [41]. QS can promote the growth and colonization of bacteria, and quorum sensing can increase the relative abundance of *Bifidobacterium* in the intestine and improve the body's immunity. On the other hand, according to the Asahara's study, we think that the increase in the relative abundance of *Bifidobacterium* may inhibit the toxins of some pathogenic bacteria, reduce the risk of infection by pathogenic bacteria. From the above two aspects, it can be considered that the quorum sensing of *Bifidobacterium*may improves human health.

*Lactobacillus* maintains the microecological balance of the intestine and vagina. The LuxS/AI-2 system in *Lactobacillus plantarum* and the production of bacteriocin regulate the QS system [42]. *Lactobacillus plantarum* inhibits the pathogens associated with wound infections through QQ (*Pseudomonas aeruginosa* PAO1/ATCC 27853, methicillin-resistant *Staphylococcus aureus* ATCC 43300, and hospital-derived strains) [43]. *L. plantarum* inhibits the production of *P. aeruginosa* AHLs, and the virulence factors controlled by these signaling molecules, including elastase and biofilms. When mouse models with burns infected with *P. aeruginosa* were treated with *L. plantarum*, it inhibited the colonization in *P. aeruginosa* [44]. In *in vivo* assays, the AI-2 activity of enterohemorrhagic *Escherichia coli* was significantly inhibited following the administration of *Lactobacillus acidophilus* in weaning pigs, demonstrating that *L. acidophilus* regulates the intestinal microflora of pigs through QQ [45]. In other studies, *Streptococcus mutans* was shown to promote dental caries, and its growth and virulence are inhibited by *Lactobacillus* sp. (including *L. casei subspecies casei* (ATCC 393), *L. reuteri* (ATCC 23272), *L. plantarum* subsp. *Plantarum* (ATCC 14917) and *L. salivarius* (ATCC 11741)) [46]. *Lactobacillus* may promote self-growth by enhancing QS, inhibiting the proliferation of pathogenic bacteria, and subsequently improving human health.

*Clostridium difficile* is an intestinal flora in newborns. The LuxS/AI-2 system has been found in *C. difficile* where it regulates virulence gene (tcdA, tcdB, tcdE) expression [47]. *Pediococcus acidilactici* is a widely used probiotic. The lactic acid produced by *Pediococcus acidilactici* M7 strains inhibits short-chain HSL production and swarming-swimming-twitching motility, elastase, protease, pyocyanin, and biofilm production in *Pseudomonas aeruginosa* [48].

*Escherichia coli* Nissle is a widely used probiotic for the treatment of common gastrointestinal diseases. *E. coli* Nissle produces AI-2, whilst the *luxs* gene in the AI-2 system affect the expression of proinflammatory cytokines (IFN-*γ*, IL-6, and TNF-*α*) in mouse models of acute colitis [49]. Soluble and cellular fractions of *Enterococcus* (*E.*) *faecium* CMGB16 also interfere with the adherence capacity and antibiotic susceptibility of enteropathogenic *Escherichia* (*E.*) *coli* strains [50].

### 3.2. Quorum Sensing Influences the Pathogenic Characteristics of Bacteria

Pathogens use the QS system to regulate biofilm formation, exopolysaccharide production, bacterial virulence, and motility. *Streptococcus pneumoniae* is a common pathogen that causes otitis media, sinusitis, and pneumonia. *S. pneumoniae* is colonized by the biofilm in the nasopharynx. The LuxS/AI-2 system regulates the transcript levels of lytA (which encodes an autolysin previously implicated in biofilm formation), and the transcript levels of ply (which encodes pneumococcal pneumolysin), that regulates biofilm formation in *S. pneumoniae* [51]. In further studies, cDNA microarrays were used to investigate the global gene expression of *S. pneumoniae* regulated by LuxS/AI-2. The LuxS/AI-2 QS system is necessary for biofilm formation and the colonization of the ear epithelium through its regulation of genes mediating virulence and bacterial fitness during pneumococcal biofilm formation [52].

*Pseudomonas aeruginosa*, a conditional pathogen, directly controls the expression of multiple virulence factors through the QS-related protein MvfR [53]. Moreover, *P. aeruginosa* uses the QS system to activate the expression of genes involved in the bacterial CRISPR-Cas autoimmune system, thereby increasing bacterial immunity [54].

Two types of QS systems are present in *Staphylococcus aureus*: the Agr system based on the autoinducing peptide AIP and the LuxS/AI-2 system. *S. aureus* inhibits the biofilm regulatory factor rbf through LuxS/AI-2, which in turn regulates biofilm formation [55]. *Bacillus* interferes with the QS of *S. aureus* by secreting fenvalin fengycins, preventing the colonization of *S. aureus* in the intestine. This provides direct mechanistic evidence of how probiotics can inhibit the colonization of pathogenic bacteria [56].

Pathogenic *Escherichia coli* causes bloody diarrhea and hemolytic-uremic syndrome through QS systems such as LuxS/AI-2. QS regulates biofilm and the expression of virulence factors in enterohemorrhagic *E. coli* [57, 58]. *Lactobacillus plantarum* and *Bacillus* spp., when used with EPS at an appropriate concentration (>1 mg/ml), inhibit *E. coli* ATCC35218 biofilm formation and reduce efflux pumps implicated in bacterial adhesion and antimicrobial resistance [59]. Elaeed-exopolysaccharides (r-EPS) from *Lactobacillus acidophilus* A4 similarly suppress biofilm formation through its influence on genes related to curli production (crl, csgA, and csgB) and chemotaxis (cheY) through transcriptome analysis [60]. The supernatants of *Enterococcus faecium* strains influence the growth, adhesion, and biofilm formation of enteroaggregative *Escherichia coli* (EAggEC) [61].

*Enterococcus faecalis* are Gram-positive bacteria that cause a variety of nosocomial infections of which urinary tract infections are most common. The Fsr and LuxS/AI-2 systems were identified in *E. faecalis*. The faecal induction-related gene fsrB of *E. faecalis* is associated with bacterial toxicity in rabbit models of endophthalmitis [62]. In addition, the luxS gene in the LuxS/AI-2 system is closely related to biofilm formation in *Enterococcus faecalis* [63].

*Vibrio cholerae* infection leads to severe diarrhea and even death, with epidemics persisting in many countries. The QS autoinducer of *V. cholerae*, cholera autoinducer 1 (CAI-1), AI-2, and the central regulator LuxO influence the expression of EPS, which in turn affects bacterial biofilm formation [64]. *Vibrio cholerae* was inhibited by probiotics including *Lactococcus lactis* in mice models through its ability to produce lactic acid in the intestine. These results highlight the ability of probiotics to transform the intestinal environment to induce the resistance to pathogenic bacteria colonization [65].

*Salmonella typhimurium* infection leads to typhoid fever, an intestinal infectious disease. *S. Typhimurium* responds to two different AHL QS signals (C6-AHL and C8-AHL) [66]. AHLs are recognized by SdiA, believed to be a sensor of AHLs produced by other bacteria. The QS regulator SdiA in *S. typhimurium* regulates the outer membrane protein Rck [67].

## 4. Quorum-Sensing Substances from Natural Products

Natural products have been studied for their therapeutic value in traditional medical practice, but interests in their ability to regulate bacterial microecology have intensified. The use of QS agents is now considered an effective mechanism to treat microbial-related diseases through their ability to inhibit antibiotic resistance.

The rates of drug-resistance nosocomial pathogenic bacterial infections are increasing annually. Bacterial infection can be inhibited by preventing QS. We now summarize the QS agonists or antagonists from natural sources to further understand these effects.

### 4.1. Regulation of Natural Products in Bacterial Biofilms

Biofilm is a self-protecting form of bacterial colonization that occurs on the surface of host receptors to resist adverse growth. QS plays a vital role in the formation of bacterial biofilms. The QS between bacteria promotes the formation of probiotic biofilms and improves the resistance of strains, promotes bacterial growth, and enhances probiotic effects. In addition, the bacterial QS system can be used to inhibit biofilm formation in pathogenic bacteria, reducing the risk of infection and avoiding bacterial resistance.

In screening for QS active substances, studies have paid more attention to medicinal plant resources. The methanol extract of *Psoralea corylifolia L.* reduces the QS activity of pathogenic bacteria (*Pseudomonas aeruginosa*, *Serratia marcescens*, and *Aeromonas hydrophila*). Further molecular docking results show that bakuchiol of the extracts binds to QS-related proteins and inhibits biofilm formation [68]. Methanolic extracts of the Indian medicinal plant *Cuminum cyminum* also promote the loosening of the biofilm architecture and powerfully inhibits *in vitro* biofilm formation in *Pseudomonas aeruginosa* PAO1, *Proteus mirabilis*, and *Serratia marcescens* at sub-MIC levels. The results of molecular docking analysis showed that methyl eugenol (ME) in *C. cyminum* mediates quorum-sensing inhibitors (QSI) activity [69]. *Liriodendron tulipifera*, *Aralia spinosa*, and *Quercus alba* are medicinal plants native to the southern United States. These plant extracts inhibit the biofilm formation in *Staphylococcus aureus* [70].

Food materials are rich in natural products. The screening process of natural QS substances in natural products derived from food has attracted attention due to their known safety. Corilagin from the fruit of *Terminalia chebula Retz* is widely used as a food supplement in China. The extract of *T. chebula* and its phenolic acid, corilagin, show antivirulence activity against *Staphylococcus aureus*. Corilagin also reduces the transcription of genes related to quorum sensing (staphylococcal accessory regulator A, intercellular adhesion accessory gene regulator A, and RNAIII) [71]. Water-soluble extracts from the North American cranberry could inhibit *V. cholerae* biofilms during the development/maturation stage by reducing the biofilm matrix production and secretion [72]. The phenolic extracts of the edible plant *Rubus rosaefolius* inhibit pigment production, cluster movement, and biofilm formation in *Chromobacterium violaceum* 12472 [73].

Small molecule compounds have been isolated from medicinal plants that inhibit the QS system. Curcumin is the main active ingredient of *Curcumae longae* L. and *Arhizoma erubescens* (Wall.) Schott inhibits biofilm formation and movement in *Escherichia coli*, *Pseudomonas aeruginosa* PAO1, *Proteus mirabilis*, and *Serratia marcescens*, and has QSI effects on a variety of bacteria, acting as a broad-spectrum QQ [74]. Quercetin, apigenin, and luteolin inhibit biofilm formation in *Chromobacterium violaceum* (strain ATCC 12472). These compounds are effective against *Pseudomonas aeruginosa* PAO1 [75]. A halogenated furanone (C_5_H_3_BrO_3_) isolated from the Australian edible macroalga *Delisea pulchra* inhibits the formation of biofilms in *P. aeruginosa* sensing systems. Green-fluorescent protein (GFP) penetrates microcolonies and blocks signaling and QS in biofilms [76]. Coumarin has an aromatic odor and is widely distributed in plants. Coumarin and its derivatives show extensive QS activity and both coumarin and umbelliferone inhibit biofilm formation in *E. coli* O157:H7. Transcriptional analysis showed that coumarin inhibits *curli* and the motor gene of *E. coli* O157:H7. In addition, esculetin inhibits Shiga-like toxins *stx2* in *E. coli* O157:H7 and attenuates its virulence in nematodes *Caenorhabditis elegans* [77].

### 4.2. Regulation of Natural Products on Bacterial Virulence

Bacterial virulence factors cause bacterial disease. Recent studies have shown that QS controls the expression of bacterial virulence. Increasing attention has been paid to the use of QS for inhibiting the virulence of bacteria.

In natural products extracts, *Forsythia suspense* (thunb.) Vahl could inhibit QS-regulated virulence factors production and biofilm formation in *P. aeruginosa* in a concentration-dependent manner. Elastase activity and pyocyanin production were inhibited at a maximum of 40.97% and 47.58% when *P. aeruginosa* was grown in the presence of 0.25 g/mL water extract [78]. Distillation products from a medicinal formulation (*Artemisia argyi* Levi.et Vant., *Dictamnus dasycarpus* Turcz., and *Solanum nigrumL.*) markedly attenuated the production of virulence factors of *Pseudomonas aeruginosa*, including phenazine pyocyanin, siderophore pyoverdine, and biofilm formation. Distillation products inhibit binding of the PQS receptor MvfR to the corresponding pqsA promoter to inhibit the QS system, and the Pseudomonas quinolone-signaling (PQS) system [79]. Methanol-soluble extracts from *Ganoderma lucidum* inhibited QS in *Chromobacterium violaceum* CV026 [80]. *Syzygium aromaticum* extracts inhibited QS-regulated phenotypes in *Pseudomonas aeruginosa* PA01, including the expression of virulence factor (pyocyanin) [81]. Purified fractions (EA) from the leaf extracts of *Syzygium cumini* (*L.*) Skeels, a traditional Indian drug used to treat diabetes, inhibit biofilm formation and virulence factors in *P. aeruginosa* and *S. aureus.* Bioactive compounds detected by GC-MS were shown to interact with RhlG/NADP active-site complex (PDB ID: 2B4Q), LasR-TP4 complexes (PDB ID: 3JPU), and Pseudaminidase (PDB ID: 2W38) [82]. Extracts of three Indian medicinal plants, *Astilbe rivularis*, *Fragaria nubicola*, and *Osbeckia nepalensis*, show a dose-dependent inhibition of violacein production in *Chromobacterium violaceum* MTCC 2656 and pyocyanin in *Pseudomonas aeruginosa* MTCC 2297 [83]. *Melicope lunuankenda* (Gaertn.) T. G. Hartley, an endemic plant in Malaysia, interferes with violacein production in *Chromobacterium violaceum* CV026; reduces bioluminescence expression in *E. coli* [pSB401], disrupts pyocyanin synthesis, swarming motility and the expression of *lecA::lux* in Pseudomonas aeruginosa PAO1 [84]. *Musa acuminata* peel (MAM) is known for its healing and antiseptic properties and is common in South Asia and Africa. MAM significantly inhibits the biofilms of *Pseudomonas aeruginosa* and inhibits QS-mediated virulence. The anti-QS activity of MAM is mediated by 5-hydroxymethylfurfural [85]. Water extracts of *Nymphaea tetragona* significantly lower the levels of violacein of *Chromobacterium violaceum*. The swarming motility of *P. aeruginosa* was also inhibited [86].

Low concentrations of honey inhibit the expression of *lsrA/tnaA*, a gene induced by enterohemorrhagic *Escherichia coli*, that inhibits the expression of *curli*, a biofilm-forming gene, and reduces the adhesion and virulence of *E. coli* in intestinal epithelial cells [87]. Liquorice, celery, cayenne pepper, and aniseed show high anti-QS potential, the chief active compound from celery, isolated and identified as 3--butyl-4,5-dihydrophthalide (sedanenolide) [88]. Extracts of *Castanea sativa* leaves inhibit the expression of the QS regulatory gene (*agr*) in *Staphylococcus aureus* and inhibit the virulence of *Staphylococcus aureus* [89].

Some small molecules also influence QS activity. Punicalagin, an active ingredient in the Chinese herbal medicine *Punica granatum* L., downregulates the expression of *Serratia marcescens* virulence-related genes and the expression of QS-related genes (*sdiA* and *srgE*), reducing the invasion of colon cells by *S. marcescens* [90]. Trans-cinnamaldehyde (CA) and salicylic acid (SA) in *Cinnamomum cassia* Presl also effectively downregulate both the las and rhl QS systems and significantly reduce virulence phenotypes at both the transcriptional and extracellular levels in *P. aeruginosa* PAO1 [91]. Zeaxanthin, an active constituent of Chinese herbal medicine *Lycium barbarum* L., reduces the expression of the virulence factors of *Pseudomonas aeruginosa* by QS [92]. Sodium houttuyfonate, derived from the Chinese herbal medicine *Houttuynia cordata* Thunb., inhibits the production of virulence factors (pyocyanin) in *Pseudomonas aeruginosa* through downregulating the expression of the AHL biosynthesis gene *lasl* and the transcription factor lasR, exerting inhibitory effects related to biofilm formation [93]. Tea polyphenols inhibit the production of the *Chromobacterium violaceum* 12472 virulence factor violacein with an almost 98% reduction at 3.125 mg/mL. Violacein is directly related to AHL and exhibited inhibitory effects on virulence phenotypes (proteolytic activity, elastase, swarming motility, and biofilm formation) regulated by QS in *Pseudomonas aeruginosa* [94]. Quercetin, a flavonol found in onions, interferes with the production of violacein and swarming motility in *Chromobacterium violaceum* [95].

Many QS actives are currently found in natural products. QS actives mainly exist in the methanol extraction part of the extract. In addition, in small molecule actives with well-defined structures, we find that these compounds often contain aldehyde and ketone structures and that these small molecule compounds are often also active ingredients of the natural product. The structural analogs of these compounds can guide the discovery of QS active substances. The composition of most QS actives is complex and further screening of single and high-efficiency QS active substances is required.

## 5. Conclusions

Bacteria regulate gene expression in a population-dependent manner by sensing the levels of autoinducer molecules that modulate the external environment. QS plays a vital role in bacterial biofilm formation, virulence factor regulation, and antibiotic formation. Natural compounds regulate QS between bacteria by controlling gene expression, biofilm formation, and the production of virulence factors.

To date, progress has been made regarding the development of drugs based on QS. For example, libraries of nonnative AHLs have been designed and synthesized. Modulators of the R protein of *A. tumefaciens*, *P. aeruginosa*, and *V. fischeri* have been identified. The structural characteristics of these proteins should lead to the development of related drugs [96]. Altering the structure of these derivatives can overcome the problem of species specificity. A synthetic analog (QS0108) was synthesized based on the molecular structure of *P. aeruginosa*, which when conjugated to ciprofloxacin, significantly inhibited the nascent and mature biofilms of *P. aeruginosa* [97]. A1-2 analogs bearing aromatic rings have also been synthesized in C-5 aromatic substituted furanones that inhibit biofilm formation and virulence production in *P. aeruginosa* [37].

In addition, key proteins in the QS system have been studied. Nonnatural triaryl series LasR ligands can be screened through the combination of structure-activity relationships and computational modeling. Strategies to induce LasR antagonism were proposed, and the key structural characteristics of this ligand class were defined [98].

Studies have confirmed that RNAIII-inhibiting peptide (RIP) prevents experimental *Staphylococcal* (*methicillin-resistant Staphylococcus aureus* and *Staphylococcus epidermidis*) infections. These act as effective staphylococcus inhibitors. Virtual screening of RIP-based pharmacophores was performed through a database, and 2′,5-di-O-galloyl-d-hamamelose (hamamelitannin) was selected as a nonpeptide analog of RIP. Further studies confirmed that hamamelitannin reduces the colonization of memeticillin-resistant *Staphylococcus aureus* and *Staphylococcus epidermidis* strains in mice. Hamamelitannin can be used as an inhibitor of staphylococcal infections. An in-depth investigation of quorum-sensing control may lead to the development of such novel antipathogenic drugs [99].

Natural products are lead compounds in drug discovery. Natural products regulate QS and alter the group behavior of some bacteria, but the material basis and mechanisms of action that alleviate human disease remain unclear, and its specific target and mode of action for regulating bacterial QS are poorly defined. Most natural product QS actives are complex extracts, not single compounds. The specific mechanism of these components in the QS system is still unclear. Which of these complex components has better activity on the QS system needs further research. Moreover, how natural products with vastly different structures and physicochemical properties act on QS genes remains unclear. Therefore, we believe that research in this field should focus on the screening of high-efficiency QS active substances from natural product, explore their influence on key nodes of QS, and clarify the specificity of active substances and their targets.

At present, studies on bacterial biofilms and virulence factors by natural products have received increasing attention, such as controlling the formation of biofilms and the production of virulence factors by affecting the expression of related genes and through competitive binding with signaling molecules. Whether natural products participate in other biological aspects of QS, such as the formation of bacterial antibiotics, DNA transfer, and bioluminescence, are less well studied. Moreover, studies on the natural products of QS remain in their. Most studies have focused on *in vitro* experiments, but the natural products on QS must be assessed in complex organisms. At present, both quorum sensing and quorum quenching are research hotspots. In the future, we should pay attention to the application of QS inhibitors and QS agonists. The widespread application of QS to clinical therapeutics and production and living is the focus of future research.

## Figures and Tables

**Table 1:** Several common quorum-sensing systems.

Signal molecule of QS system	Core structure	Structure example	Pathway	Key protein	Bacteria
AHL	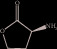	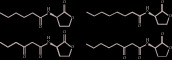 [14]	luxslR	LuxI, LuxR	*Vibrio fischeri* [15]
			LasIR-RhlIR	Lasl,LasR,RhlI,RhlR	*Pseudomonas aeruginosa* [16]
			ExpIR	ExpR,ExpI	*Erwinia carotovora* [16],*Dickeya dadantii* [17]
			SmaI/SmaR	PhoR,PhoB	*Serratia* sp. [18]

AIP		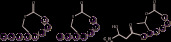 [19]	Agr	Agr A,Agr B,Agr C	*Staphylococcus aureus* [20],*Listeria monocytogenes* [21]
			Fsr	FsrA,FsrB,FsrC	*Enterococcus faecalis* [22],*Staphylococcus aureus* [23]
			Competitive quorum-sensing system	RapB,RapC, ComP, ComQ	*Bacillus subtilis* [16]
			Cytolysin quorum-sensing system	CylA, CylB,CylM	*Enterococcus faecalis* [22]
			Phr-peptide regulatory system	Opp,SecA	*Bacillus subtilis* [23]
			Extracellular protease processed AIP	plcR,OPP	*Bacillus cereus* [20]

AI-2	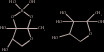	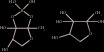 [24]	LuxS/AI-2	Pfs,LuxS	*Vibrio harveyi* [25],*Streptococcus agalactiae* [26], *Haemophilus parasuis* [27]
			Lsr	LsrK,LsrR	*Escherichia coli* [28]

Others			AI-3	ClpXP,Fis	Enterohemorrhagic *Escherichia coli* [29]
		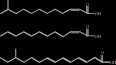 [30]	DSF	RpfC,RpfG	*Xanthomonas campestris* [30],*Xanthomonas oryzae* [31]
		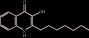 [30]	PQS	PqsR	*Pseudomonas aeruginosa* [32]
		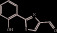 [33]	IQS	AmbBCDE	*Pseudomonas aeruginosa* [33]
